# Sustainability of a Clinical Decision Support Intervention for Outpatient Care for Emergency Department Patients With Acute Pulmonary Embolism

**DOI:** 10.1001/jamanetworkopen.2022.12340

**Published:** 2022-05-16

**Authors:** David R. Vinson, Scott D. Casey, Peter L. Vuong, Jie Huang, Dustin W. Ballard, Mary E. Reed

**Affiliations:** 1 The Permanente Medical Group, Oakland, California; 2 Kaiser Permanente Division of Research, Oakland, California; 3 The Kaiser Permanente CREST Network; 4 Department of Emergency Medicine, Kaiser Permanente Roseville Medical Center, Roseville, California; 5 Department of Emergency Medicine, UC Davis Health, University of California, Davis, Sacramento; 6 Department of Emergency Medicine, Kaiser Permanente Modesto Medical Center, Modesto, California; 7 Department of Emergency Medicine, Kaiser Permanente San Rafael Medical Center, San Rafael, California

## Abstract

**Importance:**

Physicians commonly hospitalize patients presenting to the emergency department (ED) with acute pulmonary embolism (PE), despite eligibility for safe outpatient management. Risk stratification using electronic health record–embedded clinical decision support systems can aid physician site-of-care decision-making and increase safe outpatient management. The long-term sustainability of early improvements after the cessation of trial-based, champion-led promotion is uncertain.

**Objective:**

To evaluate the sustainability of recommended site-of-care decision-making support 4 years after initial physician champion-led interventions to increase outpatient management for patients with acute PE.

**Design, Setting, and Participants:**

This retrospective cohort study was conducted in 21 US community hospitals in an integrated health system. Participants included adult patients presenting to the ED with acute PE. Study sites had participated in an original decision-support intervention trial 4 years prior to the current study period: 10 sites were intervention sites, 11 sites were controls. In that trial, decision support with champion promotion resulted in significantly higher outpatient management at intervention sites compared with controls. After trial completion, all study sites were given continued access to a modified decision-support tool without further champion-led outreach. Data were analyzed from January 2019 to February 2020.

**Exposures:**

ED treatment with a modified clinical decision support tool.

**Main Outcomes and Measures:**

The main outcome was frequency of outpatient management, defined as discharge home directly from the ED, stratified by the PE Severity Index. The safety measure of outpatient care was 7-day PE-related hospitalization.

**Results:**

This study included 1039 patients, including 533 (51.3%) women, with a median (IQR) age of 65 (52-74) years. Nearly half (474 patients [45.6%]) were rated lower risk on the PE Severity Index. Overall, 278 patients (26.8%) were treated as outpatients, with only four 7-day PE-related hospitalizations (1.4%; 95% CI, 0.4%-3.6%). The practice gap in outpatient management created by the earlier trial persisted in the outpatient management for patients with lower risk: 109 of 236 patients (46.2%) at former intervention sites vs 81 of 238 patients (34.0%) at former control sites (difference, 12.2; [95% CI, 3.4-20.9] percentage points; *P* = .007), with wide interfacility variation (range, 7.1%-47.1%).

**Conclusions and Relevance:**

In this cohort study, a champion-led, decision-support intervention to increase outpatient management for patients presenting to the ED with acute pulmonary embolism was associated with sustained higher rates of outpatient management 4 years later. The application of our findings to improving sustainability of practice change for other clinical conditions warrants further study.

## Key Points

**Question:**

Were the outcomes associated with a physician champion-led, electronic health record–embedded clinical decision support intervention for risk-stratifying adults presenting at the emergency department (ED) with acute pulmonary embolism sustained 4 years after initial promotion?

**Findings:**

In this cohort study of 1039 patients across 21 EDs, outpatient management increased significantly compared with prior practices in former control EDs. Former intervention sites continued to outperform former controls in managing acute pulmonary embolism among patients with low risk.

**Meaning:**

These findings suggest that this champion-led, clinical decision support intervention was associated with sustained practice change in identifying and safely discharging patients with low-risk pulmonary embolism.

## Teaser

This cohort study evaluates the sustainability of recommended site-of-care decision-making support 4 years after an initial intervention to increase outpatient management of acute pulmonary embolism among patients presenting to the emergency department (ED).

## Introduction

Patients with low-risk pulmonary embolism (PE) can be safely treated without hospitalization.^1,2,3,4,5,6,7,8^ Outpatient management better stewards health care resources and helps patients avoid the cost, inconvenience, and risk associated with hospitalization.^7,9,10^ Society guidelines recommend outpatient management for patients with low risk.^11,12,13,14^ However, US physicians have been slow to embrace these recommendations.^15^ Operational barriers include difficult access to pharmacotherapy and timely follow-up. Physician barriers include discomfort with the unfamiliar, aversion to complexity, and concern about medicolegal risks.^16^

In 2014, we implemented the electronic Support for Pulmonary Embolism Emergency Disposition (eSPEED) trial to help overcome physician barriers in a health care system with ready access to pharmacotherapy and timely follow-up.^7^ Intervention emergency departments (EDs) had a web-based clinical decision-support system (CDSS) integrated into the electronic health record (EHR) to provide evidence-based, risk-stratified recommendations to guide site-of-care decision-making for emergency physicians treating ED patients with acute PE. Onsite peer champions provided physician education, CDSS promotion, audit and feedback, and role modeling. During an 8-month intervention period, outpatient management safely increased in intervention EDs without any change in controls.^7^

**Figure 1.**
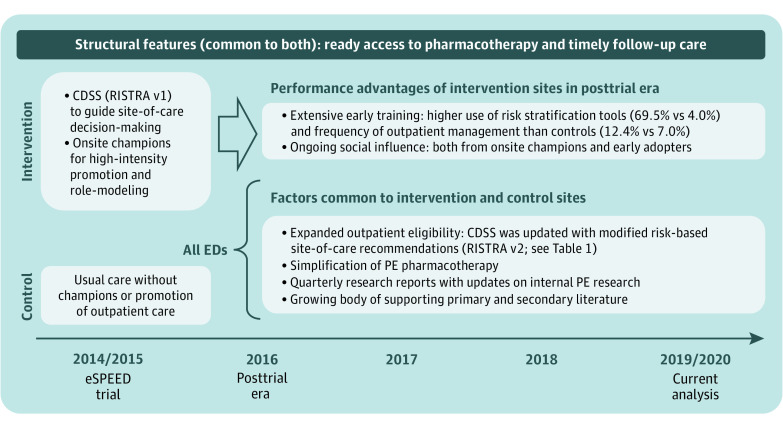
Timeline of Factors Facilitating Outpatient Management of Emergency Department Patients with Acute Pulmonary Embolism (PE) in Intervention and Control Sites The electronic Support for Pulmonary Embolism Emergency Disposition (eSPEED) trial assigned 10 emergency departments (EDs) to the intervention group and 11 to the control group based on the presence of an onsite study champion. The intervention included use of a web-based clinical decision support system (CDSS) for risk stratification called RISTRA integrated into the ED navigator of the electronic health record. RISTRA version 1 (v1) was introduced at intervention sites in late 2014 and v2 was introduced to intervention sites in 2016 and control sites in 2017.

EHR-embedded decision support can improve physician decision-making,^17,18^ even in busy EDs.^17,19^ But effect durability has not been well studied. To be sustainable, practice innovations must continue their impact in the absence of their initial promotion and must adapt to changing conditions and evidence.^20,21^ With this in mind, we updated CDSS site-of-care recommendations in 2016 to reflect our trial results, broadening outpatient eligibility without changing hospitalization indications. We gave decision-support access to control EDs with a 1-hour educational and training session. However, after eSPEED, no EDs received structured promotion, and control EDs were never assigned a champion (Figure 1).

In this 14-month retrospective cohort study in 2019 to 2020 across 21 EDs, we compared frequencies of outpatient management between former intervention and control EDs. We hypothesized that performance gains experienced earlier by intervention EDs would be sustained and that posttrial interventions would be associated with increased outpatient management in both ED groups. Lessons from this study may assist clinicians in providing the level of care that matches patient risk for those with acute PE.

## Methods

This cohort study was approved by the Kaiser Permanente Northern California (KPNC) institutional review board with a waiver for the requirement for written informed consent because of the observational nature of the study in the course of usual care. This study followed the Strengthening the Reporting of Observational Studies in Epidemiology (STROBE) reporting guideline.

### Design and Setting

This Sustained Effects (SUS-EFX) Study was a retrospective cohort study from January 2019 to February 2020 of all 21 community-based EDs of KPNC, an integrated health system that serves more than 4.5 million members who represent the surrounding racial, ethnic, and socioeconomic diversity of California.^22^ KPNC uses a comprehensive EHR, including outpatient, emergency, inpatient, laboratory, imaging, and pharmacy history.

The included 21 EDs are staffed by board-certified (or board-eligible) emergency physicians. In KPNC, PE is usually diagnosed in the ED; patients diagnosed in the clinic setting are commonly referred to an ED for definitive care.^1^ The system recommended direct oral anticoagulants (DOACs) for the treatment of most patients with acute PE (eAppendix 1 in the Supplement). Patients received timely follow-up^23^ and had access to anticoagulants with long-term monitoring by a pharmacy-led telephone-based anticoagulation management service,^24,25,26^ which contacts patients for education shortly after ED discharge. More information on ready access to pharmacotherapy and close follow-up is provided in eAppendix 2 in the Supplement.

We used evidence-based principles to design and build a web-based, EHR-integrated CDSS that we call RISTRA-PE (for risk stratification).^27,28,29,30^ It is accessible within the ED navigator of the EHR. Activation of RISTRA-PE after diagnostic confirmation of PE is physician-driven and entirely voluntary. There is no prompt or best-practice alert. RISTRA-PE includes an autopopulating version of the validated PE Severity Index (eTable 1 in the Supplement)^8,31,32,55^ with risk-based recommendations to inform site-of-care decision-making (Table 1) and outpatient exclusion criteria modeled after the Canadian criteria and Hestia clinical decision rule (eFigure in the Supplement).^34,35^ Our site-of-care recommendations were designed to be assistive, not directive (eAppendix 3 in the Supplement).^36^ The presence of right ventricular dysfunction was among our exclusion criteria, given the associated increase in short-term risk, even among patients with low risk.^37,38,39,40^ As with the 2016 CHEST guidelines,^41^ we did not recommend routine assessment of right ventricular dysfunction.

**Table 1.** Changing Risk-Based Site-of-Care Recommendations for Emergency Department Patients With Acute Pulmonary Embolism

Pulmonary Embolism Severity Index Score, points (Class)^d^	RISTRA-PE version 1^a^		RISTRA-PE version 2^b^		
	Approximate 30-d all-cause mortality, %^e^	Initial care recommendation	All-cause mortality, %^c^		Initial care recommendation
			7-d	30-d	
≤64 (I)	<2	Outpatient management is often possible	0	0	Outpatient management is often appropriate
65-85 (II)	<2	Outpatient management is often possible	<1	<1	Outpatient management is often appropriate
86-105 (III)	5	Inpatient care is often indicated	<1	3	Outpatient management may be possible
106-125 (IV)	10	Inpatient care is often indicated	<1	5	Outpatient management may be possible
≥126 (V)	20	Inpatient care is often indicated	5	13	Inpatient care is often indicated

Abbreviations: ED, emergency department; RISTRA-PE, Risk Stratification for Pulmonary Embolism.

^a^ Launched September 2014 and promoted at 10 intervention EDs.

^b^ Accessible March 2017 to all 21 EDs.

^c^ Estimates based on internal data from the electronic Support for Pulmonary Embolism Emergency Disposition^7^ trial and associated studies.^56^

^d^ The Pulmonary Embolism Severity Index is presented in eTable 1 in the Supplement.

^e^ Estimates based on the Pulmonary Embolism Severity Index literature as of 2014.^7^

The 8-month eSPEED intervention ran from September 2014 through April 2015.^7^ Site assignment was not randomized. The 10 EDs that already had an onsite clinical champion received RISTRA-PE access (the original version 1) with champion-led promotion. These constituted the intervention sites. The other 11 EDs did not have an onsite champion and served as concurrent controls. Champions provided iterative physician education, personalized audit and feedback (eAppendix 4 in the Supplement), monthly emails reporting facility enrollment rates that commended leading and new enrollers, and small incentives for each physician’s first 3 enrollments. Champions also served as ED role models.^7,42,43^ Although CDSS use was unprompted, uptake across intervention sites was high (68.9%).^44^ Outpatient PE management, broadly defined as discharge home from the ED or an outpatient observation unit within 24 hours,^45^ increased at 10 intervention EDs, from 17.8% of patients receiving outpatient PE management to 28.3% of patients receiving outpatient PE management (a relative 59% increase). Restricting analysis to only ED discharges, the frequency increased from 7.8% of patients discharged home to 12.4% of patients discharged home. There was no increase in discharge home at 11 control EDs: 8.0% of patients were discharged home during the preintervention period, and 7.0% of patients were discharged home in the postintervention period.^7^

### Posttrial Interventions

After completion of the eSPEED trial, we first updated RISTRA-PE’s risk-specific, site-of-care recommendations using trial results (starting in April 2016), expanding the scope of outpatient eligibility without changing outpatient exclusion criteria (Table 1; eFigure in the Supplement). Cycling internal study results back into practice-change interventions is a learning health system goal and a requirement for sustainability.^46,47^ We subsequently provided access to RISTRA-PE version 2 across all 21 EDs, including controls, starting in February 2017 (Figure 1), introducing the tool with an emailed set of educational slides followed by an hour-long in-person educational presentation, part of a required 1-day educational forum with nearly 100% attendance. Champions continued to provide patient care at intervention EDs and may have continued to exert social influence (along with early adopters) among their immediate peers.^48,49,50,51^ However, champions provided no structured promotion of RISTRA-PE version 2: no further emails, departmental presentations, enrollment incentives, or audit and feedback were provided. There was minimal crossover of emergency physicians (<2%) working an occasional shift outside their home EDs, eg, intervention physicians at control sites.

Second, we disseminated quarterly research reports to the ED physicians with updates of our ongoing PE studies (Figure 1).^7,23,32,33,36,52,53,54,55,56^ Continued research from our own practice setting on the safety and effectiveness of outpatient management for patients with acute PE may have helped communicate that this was becoming a systemwide standard of care.^57^

Third, the medical group switched pharmacotherapy recommendations from warfarin to DOACs in 2016 in concert with CHEST guidelines.^41^ While DOACs alone may be insufficient in shifting ED site-of-care practices,^58^ simplifying pharmacotherapy might have a supportive association in systems already primed for outpatient care.

### Study Population

In the SUS-EFX study, we included health plan members aged 18 years or older with a primary diagnosis of PE in the ED accompanied by positive results for PE in computed tomography (CT) or scintigraphy imaging (either in the ED or within the prior 12 hours) from January 2019 through February 2020. We used validated natural language processing algorithms to identify positive results in CT pulmonary angiography and ventilation/perfusion scintigraphy.^1^ Patients with any of the following were excluded: a diagnosis of acute venous thromboembolism in the previous 90 days, using anticoagulants at the time of diagnosis (or an elevated ED international normalized ratio >2.0), lack of adequate health plan membership in the prior 12 months (as this affects completeness of medical history), leaving the ED against medical advice, absence of any documented ED vital signs (precluding calculation of the PE Severity Index), or known pregnancy.

### Data Collection and Study Outcomes

We obtained study demographic and clinical variables directly from the health system’s EHR using automated electronic data extraction. Race and ethnicity were self-reported and included to demonstrate that the diversity of the cohort reflects the population of northern California. We used the validated 11-variable PE Severity Index (eTable 1 in the Supplement) to estimate 30-day all-cause mortality, as previously described, and to stratify our primary outcome.^7,32,54,56^ Altered mental status was the only PE Severity Index variable not reliably available. For our analysis, we assumed results were negative (eAppendix 5 in the Supplement), as other studies of the PE Severity Index have done, including the original validation studies.^59,60^ We performed manual EHR review of approximately 10% of patients (109 patients), some randomly selected and others from targeted subpopulations, to validate 2 study variables (ie, study eligibility and initial site of care) and to adjudicate the primary safety outcome (7-day PE-related hospitalization) among outpatients, along with their 30-day all-cause mortality (eAppendix 6 in the Supplement).

Our primary study outcome was outpatient management, defined as discharge to home directly from the ED. We compared our results by original eSPEED trial assignment (intervention vs control) to evaluate the association of earlier trial assignment with recent site-of-care practices, stratified by risk classification based on the PE Severity Index. Our primary safety outcome for outpatients was 7-day hospitalization for PE-related signs, symptoms, or interventions, defined a priori and used in earlier studies (eAppendix 7 in the Supplement).^7,56^ We used claims data to identify hospitalizations outside the health system. We also measured mortality using the health system mortality database that links to the Social Security death master file and the California State Department of Vital Statistics. State mortality reports from 2020 were not yet available.

### Statistical Analysis

We used Wilcoxon nonparametric tests for continuous variables and χ^2^ tests for categorical variables to compare patient characteristics between intervention and control EDs. We reported the frequency of outpatient management and compared the difference between intervention and control EDs overall. To account for potential confounders, we compared the results between intervention and control sites stratified by the PE Severity Index, as it guided our site-of-care recommendations (Table 1) and is associated with 30-day all-cause mortality.^54^ We planned to adjust for other covariates (beyond those of the PE Severity Index) if any were found to be significantly different on bivariate analysis. We considered a 2-tailed *P* < .05 to be significant. We reported the incidence of 7-day PE-related hospitalization among those managed as outpatients and 30-day mortality among all patients. Since we did not have complete state-reported mortality data for patients diagnosed during the last 3 months of the study period (December 2019 to February 2020), we performed a sensitivity analysis of mortality outcomes by excluding these months. The number of patients during the study period determined the sample size. All analyses were conducted with SAS statistical software version 9.4 (SAS Institute). Data were analyzed from January 2019 to February 2020.

## Results

**Figure 2.**
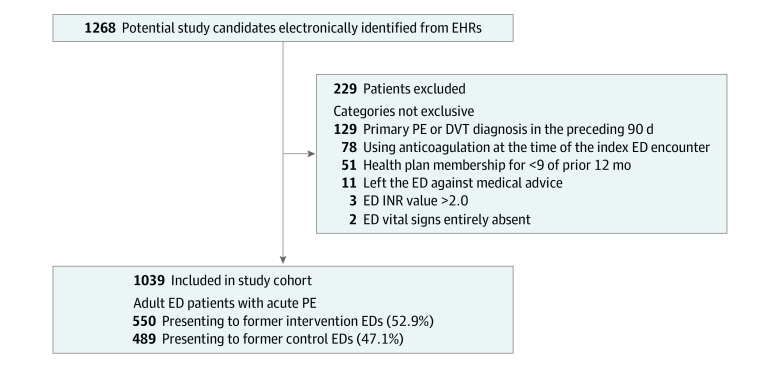
Cohort Assembly and Initial Site of Care for Adult Emergency Department (ED) Patients With Acute Pulmonary Embolism (PE) DVT indicates deep vein thrombosis; and INR, international normalized ratio.

We identified 1268 adults presenting to the ED with a PE diagnosis and positive results in a pulmonary imaging study. We excluded 229 patients, most commonly because of recent thromboembolic disease, anticoagulation, and insufficient prior health plan membership (Figure 2). The remaining 1039 patients had 1032 study-eligible encounters (7 patients had 2 eligible encounters each). In total, 553 patients (51.3%) were women, and the median (IQR) age was 65 (52-74) years (Table 2). A total of 150 patients (14.4%) were African American, 65 patients (6.3%) were Asian, 110 patients (10.6%) were Hispanic or Latinx, and 707 patients (68.1%) were White. Most patients were diagnosed using CT pulmonary angiogram (1025 patients [98.7%]). Nearly half of all patients were lower risk on the PE Severity Index (classes I-II; 474 patients [45.6%]). Overall, 278 patients (26.8%) received outpatient PE management after a median (IQR) ED length of stay of 4.7 (3.6-6.0) hours. Also, 367 patients (35.3%) were discharged home from either the ED or an outpatient observation unit, and 401 patients (38.6%) were discharged home within 24 hours of ED registration.^45^ Patients selected for outpatient care were younger, less commonly arrived by ambulance, had markedly anomalous vital signs or elevated troponin concentrations, and more commonly arrived with pre-ED imaging or were in lower-risk classes than their hospitalized counterparts (eTable 2 in the Supplement).^7,56,61^ The median (IQR) ED frequency of outpatient management was 29.0% (21.5%-31.8%), and the frequency varied widely between EDs (range, 7.1%-47.1%).

**Table 2.** ED Patients With Acute Pulmonary Embolism Stratified by Prior Trial Assignment in 2014

Characteristics	Patients, No. (%)			*P* value
	Total cohort (N = 1039)	eSPEED trial assignment ^a^		
		Intervention (n = 550)	Control (n = 489)	
Age, median (IQR)	65 (52-74)	66 (52-75)	64 (52-74)	.08
Sex				
Women	533 (51.3)	287 (52.2)	246 (50.3)	.55
Men	506 (48.7)	263 (47.8)	243 (49.7)	
Race and ethnicity^b^				
African American	150 (14.4)	85 (15.5)	65 (13.3)	.48
Asian	65 (6.3)	36 (6.6)	29 (5.9)	
Hispanic or Latinx	110 (10.6)	50 (9.1)	60 (12.3)	
White	707 (68.1)	375 (68.2)	332 (67.9)	
Other	7 (0.7)	4 (0.7)	3 (0.6)	
Comorbidities				
Chronic lung disease	275 (26.5)	145 (26.4)	130 (26.6)	.94
Cancer (active or history)	252 (24.3)	150 (27.3)	102 (20.9)	.02
Heart failure (systolic or diastolic)	43 (4.1)	26 (4.7)	17 (3.5)	.31
Arrival by ambulance	188 (18.1)	102 (18.6)	86 (17.6)	.69
Worst vital signs^c^				
Systolic blood pressure <100 mm Hg	162 (15.6)	104 (18.9)	58 (11.9)	.002
Heart rate ≥110 beats/min	278 (26.8)	165 (30.0)	113 (23.1)	.01
Respiratory rate ≥30 breaths/min	120 (11.6)	73 (13.3)	47 (9.6)	.07
Pulse oximetry <90%^d^	140 (13.5)	75 (13.6)	65 (13.3)	.87
Temperature <36 °C	19 (1.8)	10 (1.8)	9 (1.8)	.98
Diagnostic imaging, timing				
Prearrival (<12h)	107 (10.3)	55 (10.0)	52 (10.6)	.74
ED	932 (89.7)	495 (90.0)	437 (89.4)	
PE Severity Index classification^e^				
I-II (lower risk)	474 (45.6)	236 (42.9)	238 (48.7)	<.001
III-IV (intermediate risk)	393 (37.8)	199 (36.2)	194 (39.7)	
V (highest risk)	172 (16.6)	115 (20.9)	57 (11.7)	
Troponin I concentration^f^				
Within reference range	630 (60.6)	324 (58.9)	306 (62.6)	.10
Elevated	263 (25.3)	154 (28.0)	109 (22.3)	
Not performed	146 (14.1)	72 (13.1)	74 (15.1)	

Abbreviations: ED, emergency department; eSPEED, electronic Support for Pulmonary Embolism Emergency Disposition; PE, pulmonary embolism.^7^

^a^ EDs were assigned to the intervention (10 EDs) or control (11 EDs) groups based on the presence of an onsite study champion.

^b^ Race and ethnicity were self-reported. Other race and ethnicity includes Native American and Hawaiian and Pacific Islander patients.

^c^ Worst in the direction in question measured during the ED encounter. Missing values were uncommon: 0 patients were missing systolic blood pressure; 1 patient (0.1%) was missing pulse rate; 1 patient (0.1%) was missing respiratory rate; 2 patients (0.2%) were missing pulse oximetry; and 26 patients (2.5%) were missing temperature. These percentages are similar to those in the eSPEED trial. Missing vital signs were comparable between intervention and control sites.

^d^ With or without oxygen supplementation.

^e^ More information on the PE Severity Index is presented in eTable 1 in the Supplement.

^f^ Highest concentration during the ED encounter.

Patients with acute PE managed in EDs that had been former eSPEED intervention sites had a higher prevalence of cancer, anomalous vital signs, and higher mortality risk scores compared with controls. No other covariates were significantly different (Table 2). These findings supported our a priori strategy to stratify comparisons by risk classification. The 2 ED groups were comparable in terms of census, hospitalization rates, hospital bed capacity, and access to observation units, as previously reported.^7^

Overall, the frequency of outpatient management was similar between intervention and control sites (although intervention sites had patients with higher risk): 156 patients (28.4%) at intervention sites vs 122 patients (25.0%) at control sites (difference, 3.4 [95% CI, −2.2 to 8.8] percentage points; *P* = .21) (Table 3). These frequencies were higher than when measured during the eSPEED trial 4 years earlier: 12.4% in the intervention sites vs 7.0% in the control sites. When evaluated by risk strata, the intervention EDs outperformed their control counterparts among patients with lower risk: 109 patients (46.2%) vs 81 patients (34.0%) (difference, 12.2 [95% CI, 3.4 to 20.9] percentage points; *P* = .007), with no statistically significant differences among patients with higher risk (Table 3). Patients with lower risk were most likely to reflect changes in site-of-care practices because they were more commonly eligible for outpatient management and were strongly recommended for outpatient care by RISTRA-PE version 2.

**Table 3.** Frequency of Outpatient Management of Emergency Department Patients With Acute Pulmonary Embolism Stratified by 30-Day All-Cause Mortality Risk Classification

Risk group	Patients receiving outpatient management, No./total No. (%)^a^			Difference, percentage points (95% CI)
	Total cohort (N = 1039)	ED assignment during eSPEED Trial		
		Intervention (n = 550)	Control (n = 489)	
All, No. (%)	278 (26.8)	156 (28.4)	122 (24.9)	3.4 (−2.0 to 8.8)
By risk strata^b^				
Lower risk	190/474 (40.1)	109/236 (46.2)	81/238 (34.0)	12.2 (3.4 to 20.9)
Intermediate risk	74/393 (18.8)	36/199 (18.1)	38/194 (19.6)	−1.5 (−9.2 to 6.2)
Highest risk	14/172 (8.1)	11/115 (9.6)	3/57 (5.3)	4.3 (−3.6 to 12.2)

Abbreviations: ED, emergency department; eSPEED, electronic Support for Pulmonary Embolism Emergency Disposition.^7^

^a^ Outpatient management was defined as discharge home directly from the ED. Observation unit admission was categorized as hospitalization.

^b^ Thirty-day all-cause mortality risk was estimated from validated Pulmonary Embolism Severity Index classification, with lower risk including classes I and II; intermediate risk, classes III and IV; and highest risk, class V.

The incidence of 7-day PE-related hospitalization among outpatients was low (4 patients [1.4%; 95% CI, 0.4 to 3.6]): 3 patients were treated in an intervention ED and 1 patient was treated in a control ED (eTable 3 in the Supplement). Overall, 30-day all-cause mortality was 4.3%, similar to prior studies,^7,56^ and varied by site of care and risk class (eAppendix 8 and eTable 4 in the Supplement).

Physician use of RISTRA-PE version 2 for eligible ED patients was different between ED groups during the SUS-EFX study period. The tool was used for 62 of 550 physicians (11.3%) at intervention sites vs 36 of 489 physicians (7.4%) at control sites (*P* = .03). RISTRA-PE use during the SUS-EFX study period was lower at intervention sites than during the eSPEED trial several years prior: 11.3% vs 68.9%.

## Discussion

In this retrospective cohort study of 21 community-based US EDs, more than a quarter of adults with acute PE were safely discharged home after a short ED stay. During a trial conducted 4 years earlier, intervention EDs had increased outpatient management with onsite champion promotion of decision support, without an increase at control sites. The practice gap was evident 4 years later, as intervention sites continued to have increased frequency of safe outpatient management of PE among patients with lower risk, concordant with CDSS recommendations.

Posttrial interventions were similar between ED groups. However, the eSPEED trial had 2 early performance advantages that may have put intervention EDs on a different trajectory, facilitating a sustained practice gap in managing PE among patients with lower risk.^7^ First, intervention physicians had extensive early training in decision support during the eSPEED trial. Use of the tool in 69% of eligible cases helped train them in risk stratification and determination of outpatient eligibility. Early use of PE risk stratification tools may have helped internalize PE risk stratification skills, making recourse to decision support over time less necessary (hence the lower recent use rate of 11.3% at intervention sites). We had surmised as much in a prior letter: “Such evidence-based cognitive education [via CDSS use] might equip users to function without dependency on the very rules that had earlier trained their judgment.”^62^ Tool-guided training in evidence-based site-of-care decision-making may help develop a more reliable gestalt. Even with decreasing dependency on decision support at intervention sites, decision support use remained higher 4 years later than at controls: 11.3% vs 7.4% (*P* = .03).

The second performance advantage was ongoing social influence: physicians at intervention sites were exposed to local social influence by onsite champions. Advocacy by champions for new practice patterns, especially if adopted by other department peers, may have helped to create a new culture of practice, establishing new local norms of behavior, which can be transforming and long-lasting.^48,49,50,51^ Interfacility differences in degrees of cultural transformation also might have contributed to the wide facility-specific variation we observed within our own health system, variation others have also reported across the country.^15^

It is unclear whether other US EDs also experienced as sizable an increase in outpatient management as we did. ED PE studies from earlier periods (2002 to 2013) have reported mixed results.^63,64,65,66^ A more recent, broadly representative site-of-care study across 740 US EDs in 2016 to 2018 found a low frequency of outpatient management (4.1%) but did not report time trends.^15^ A large US study of claims data found that outpatient management of PE among insured patients increased from 2011 to 2018 (16% to 23%).^67^ This attests to changing practice patterns in some organizations, but the results from this study cannot be directly compared with our own (eAppendix 9 in the Supplement).

### Limitations

This study has some limitations. Our case ascertainment was incomplete, eg, we were unable to identify all patients with a clinical diagnosis of PE, such as those who lacked confirmatory pulmonary imaging. Fortunately, this population is small and likely affected intervention and control EDs equally.^56^ It is unclear which posttrial interventions may have been most directly associated with the systemwide increase in outpatient management. Given the observational study design, we cannot infer a causal relationship between posttrial interventions and physician site-of-care decision-making and cannot rule out unmeasured confounding. Incomplete documentation and missing variables that commonly beset retrospective research were mitigated regarding our outcomes, which were structured variables, reliably captured in our comprehensive EHR and not liable to be biased between ED sites. Our inability to capture unstructured documentation of mental status may have led to limited misclassification of the lower-risk cohort. However, in the eSPEED trial, altered mental status was identified in only 5% of patients, with similar rates between control and intervention sites.^7^ Additionally, our findings may not be generalizable to different populations and health care settings, especially those with less comprehensive or reliable follow-up infrastructure. However, the principal interventions we used—use of validated risk-stratification tools to provide point-of-care clinical decision support as well as enlisting onsite champions to educate, promote, and model practice change—can be readily adapted to a variety of practice settings.

## Conclusions

This cohort study found that prior gains in outpatient management of acute PE among patients in the ED fostered by champion-led CDSS promotion were associated with increased outpatient management of PE among patients with lower risk 4 years after trial cessation. Insights from the SUS-EFX study will inform how we roll out new EHR-embedded decision support for other clinical conditions, now with an eye toward sustainability without continued promotion. How early high uptake of risk-stratification tools and social influence of embedded clinical champions may contribute to the long-term sustainability of practice-change interventions for other clinical conditions warrants further study.

## Notes

Supplement. **eAppendix 1.** Anticoagulant Recommendations During the SUS-EFX Study Period **eAppendix 2.** Importance of Ready Access to Pharmacotherapy and Close Follow-up **eTable 1.** Pulmonary Embolism Severity Index **eFigure.** Example of Relative Contraindications to Outpatient Management of Emergency Department Patients With Acute Pulmonary Embolism Included in Electronic Clinical Decision Support System **eAppendix 3.** Site-of-Care Recommendations **eAppendix 4.** Method of Audit and Feedback Used in the eSPEED Trial **eAppendix 5.** Altered Mental Status Variable of the Pulmonary Embolism Severity Index **eAppendix 6.** Selective Manual Electronic Health Record Review **eAppendix 7.** Defining 7-Day Pulmonary Embolism–Related Hospitalization for Those Managed as Outpatients **eTable 2.** Emergency Department Patients With Acute Pulmonary Embolism Stratified by Direct Discharge Home vs Admission to an Observation Unit or Hospital **eTable 3.** Characteristics and Course of Emergency Department Patients With Acute Pulmonary Embolism Discharged Directly Home Who Subsequently Experienced 7-Day Hospitalization for Pulmonary Embolism–Related Concerns or 30-Day All-Cause Mortality **eAppendix 8.** Thirty-Day All-Cause Mortality of Emergency Department Patients With Acute Pulmonary Embolism **eTable 4.** Thirty-Day All-Cause Mortality of Emergency Department Patients With Acute Pulmonary Embolism Stratified by the Pulmonary Embolism Severity Index Classification **eAppendix 9.** A Large US Study of Claims Data
eReferences
