# Supplementary material for: Sustainability of a Clinical Decision Support Intervention for Outpatient Care for Emergency Department Patients With Acute Pulmonary Embolism
Source: JAMA Netw Open. 2022 May 16;5(5):e2212340. doi: 10.1001/jamanetworkopen.2022.12340 (PMC9112064; doi:10.1001/jamanetworkopen.2022.12340)
Supplement: Supplement. — eAppendix 1. Anticoagulant Recommendations During the SUS-EFX Study Period eAppendix 2. Importance of Ready Access to Pharmacotherapy and Close Follow-up eTable 1. Pulmonary Embolism Severity Index eFigure. Example of Relative Contraindications to Outpatient Management of Emergency Department Patients With Acute Pulmonary Embolism Included in Electronic Clinical Decision Support System eAppendix 3. Site-of-Care Recommendations eAppendix 4. Method of Audit and Feedback Used in the eSPEED Trial eAppendix 5. Altered Mental Status Variable of the Pulmonary Embolism Severity Index eAppendix 6. Selective Manual Electronic Health Record Review eAppendix 7. Defining 7-Day Pulmonary Embolism–Related Hospitalization for Those Managed as Outpatients eTable 2. Emergency Department Patients With Acute Pulmonary Embolism Stratified by Direct Discharge Home vs Admission to an Observation Unit or Hospital eTable 3. Characteristics and Course of Emergency Department Patients With Acute Pulmonary Embolism Discharged Directly Home Who Subsequently Experienced 7-Day Hospitalization for Pulmonary Embolism–Related Concerns or 30-Day All-Cause Mortality eAppendix 8. Thirty-Day All-Cause Mortality of Emergency Department Patients With Acute Pulmonary Embolism eTable 4. Thirty-Day All-Cause Mortality of Emergency Department Patients With Acute Pulmonary Embolism Stratified by the Pulmonary Embolism Severity Index Classification eAppendix 9. A Large US Study of Claims Data eReferences [file jamanetwopen-e2212340-s001.pdf]

Supplemental Online Content

Vinson DR, Casey SD, Vuong PL, Huang J, Ballard DW, Reed ME. Sustainability of a clinical decision support intervention for outpatient care for emergency department patients with acute pulmonary embolism. *JAMA Netw Open*. 2022;5(5):e2212340.
doi:10.1001/jamanetworkopen.2022.12340

eAppendix 1. Anticoagulant Recommendations During the SUS-EFX Study Period
eAppendix 2. Importance of Ready Access to Pharmacotherapy and Close Follow-up
eTable 1. Pulmonary Embolism Severity Index
eFigure. Example of Relative Contraindications to Outpatient Management of Emergency Department Patients With Acute Pulmonary Embolism Included in Electronic Clinical Decision Support System
eAppendix 3. Site-of-Care Recommendations
eAppendix 4. Method of Audit and Feedback Used in the eSPEED Trial
eAppendix 5. Altered Mental Status Variable of the Pulmonary Embolism Severity Index
eAppendix 6. Selective Manual Electronic Health Record Review
eAppendix 7. Defining 7-Day Pulmonary Embolism–Related Hospitalization for Those Managed as Outpatients
eTable 2. Emergency Department Patients With Acute Pulmonary Embolism Stratified by Direct Discharge Home vs Admission to an Observation Unit or Hospital
eTable 3. Characteristics and Course of Emergency Department Patients With Acute Pulmonary Embolism Discharged Directly Home Who Subsequently Experienced 7-Day Hospitalization for Pulmonary Embolism–Related Concerns or 30-Day All-Cause Mortality
eAppendix 8. Thirty-Day All-Cause Mortality of Emergency Department Patients With Acute Pulmonary Embolism
eTable 4. Thirty-Day All-Cause Mortality of Emergency Department Patients With Acute Pulmonary Embolism Stratified by the Pulmonary Embolism Severity Index Classification
eAppendix 9. A Large US Study of Claims Data
eReferences

This supplemental material has been provided by the authors to give readers additional information about their work.

© 2022 Vinson DR et al. *JAMA Network Open.*

eAppendix 1. Anticoagulant Recommendations During the SUS-EFX Study Period

Throughout the study period, guidelines from our health system recommended rivaroxaban, a direct oral anticoagulant, for the initial treatment of acute PE in patients without severe renal disease. Rivaroxaban, though not on the system's formulary, was preferred over dabigatran, as rivaroxaban avoided the need for a lead-in period of low molecular weight heparin. Dabigatran, however, was on formulary and hence less expensive for patients. For patients with active cancer, a low molecular weight heparin or a direct oral anticoagulant was recommended, depending on the location and nature of the malignancy as well as the patient's risk of bleeding. For patients with severe renal disease, warfarin was recommended with a concomitant bridge of either unfractionated or low molecular weight heparin, depending on the degree of renal insufficiency.

eAppendix 2. Importance of Ready Access to Pharmacotherapy and Close Follow-up

Ready access to pharmacotherapy and close follow-up are both prerequisites for outpatient care according to the American College of Chest Physicians (CHEST) guidelines: "In patients with low-risk PE we recommend outpatient treatment over hospitalization provided access to medications, ability to access outpatient care, and home circumstances are adequate."[1] Health systems without these structures have had to create them before implementing outpatient PE treatment pathways.[2-4]

eTable 1. Pulmonary Embolism Severity Index[5-7]

**Prognostic Variables**	**Points**[a]
**Demographic Characteristics**	
Age	+ 1 per year
Male sex	+ 10
**Comorbidities**	
Chronic lung disease (includes asthma)	+ 10
Cancer (active or history of)	+ 30
Heart failure (systolic or diastolic)	+ 10
**Clinical Findings**[b]	
Systolic blood pressure <100 mmHg	+ 30
Heart rate ≥110 beats/min	+ 20
Respiratory rate ≥30 breaths/min	+ 20
Pulse oximetry <90%[c]	+ 20
Temperature <36°C	+ 20
Altered mental status[d]	+ 60

[a] A total point score for a given patient is obtained by summing the patient's age in years and the points for each applicable prognostic variable. Point scores correspond with the following classes that estimate escalating risks of 30-day mortality: ≤65 class I, very low risk; 66-85 class II, low risk; 86-105 class III, intermediate risk; 106-125 class IV, high risk; >125 class V, very high risk.
[b] The worst vital signs during the emergency department evaluation are used
[c] With or without supplemental oxygenation
[d] Acute or pre-existing disorientation, lethargy, stupor, or coma

© 2022 Vinson DR et al. *JAMA Network Open.*

eFigure. Example of Relative Contraindications to Outpatient Management of Emergency Department Patients with Acute Pulmonary Embolism Included in Electronic Clinical Decision Support System

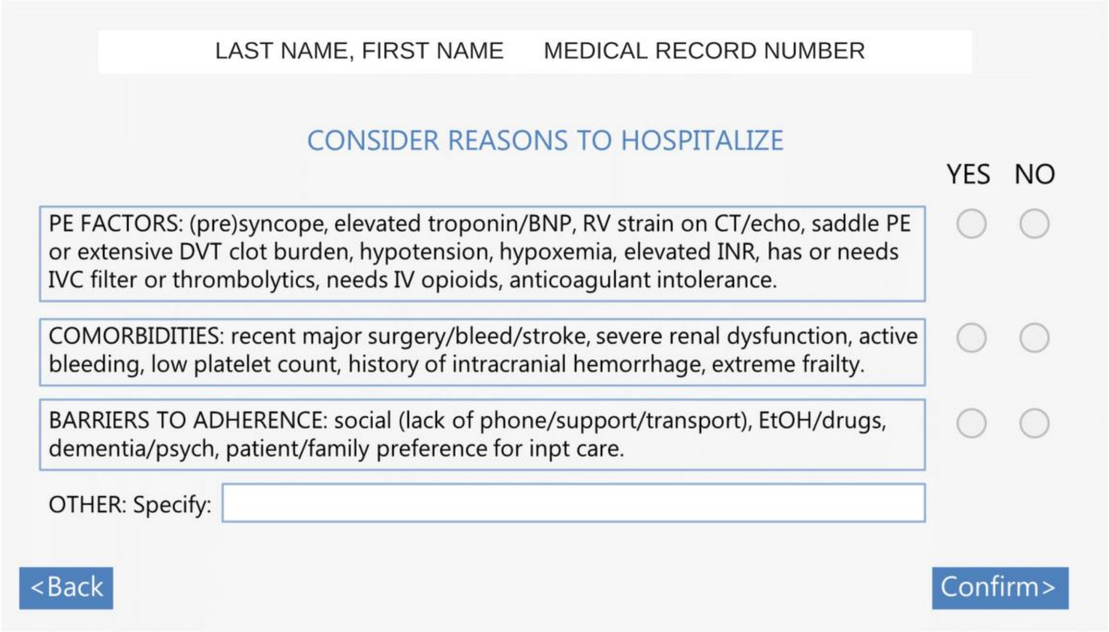

Abbreviations: BNP, B-type natriuretic peptide; CT, computed tomography; DVT, deep vein thrombosis; EtOH, ethanol; inpt, inpatient, INR, international normalized ratio; IV, intravenous; IVC, inferior vena cava; PE, pulmonary embolism; psych, psychiatry; RV, right ventricle.
This list is an expansion of the Canadian criteria and the Hestia clinical decision rule.[8,9]

eAppendix 3. Site-of-Care Recommendations

Our site-of-care recommendations were designed to be assistive, not directive, in keeping with CHEST guidelines that endorse a flexible use of prediction rules: "We consider clinical prediction rules as aids to decision-making and do not require patients to have a predefined score (e.g., low-risk [PE Severity Index] score) to be considered for treatment at home."[1,10,11] Others have noted that a strict use of the PE Severity Index to guide site-of-care decision-making would have mismatched health care resources with patient needs.[12]

eAppendix 4. Method of Audit and Feedback Used in the eSPEED Trial

Every week, the principal investigator of the eSPEED trial received a list of patients at the 10 study EDs who were probable candidates for RISTRA-PE (using criteria described previously).[13,14] He reviewed each case, confirmed eligibility, and identified among the eligible those whose physicians had used RISTRA-PE and those who had not. He sent each site champion a weekly list of their facility-specific results, which they used to provide feedback to their department.

eAppendix 5. Altered Mental Status Variable of the Pulmonary Embolism Severity Index

In prior ED PE studies in our setting, approximately 5% of patients had documented altered mental status, the variable with the highest score in the PE Severity Index (see eTable 1 above).[13,14] There were, however, no statistically significant differences in altered mental status between intervention and control EDs in the eSPEED trial.[14] The assumption that all our SUS-EFX patients lacked altered mental status will have miscategorized some actual higher-risk patients (who are unlikely to be managed as outpatients) into the lower-risk strata (just based on lower scores). This may have slightly reduced the proportion of patients categorized as lower-risk that received outpatient care. The true proportion may have been higher.

eAppendix 6. Selective Manual Electronic Health Record Review

We performed manual chart review of 109 cases. We found that computerized evaluation of study eligibility (108/109) and site-of-care categorization (106/109) were accurate. The 3 corrected site-of-care cases were found during a focused review of patients with prolonged ED length-of-stay (>12 hours). These 3 had been misclassified as ED-only cases, but in reality, had been transferred from the ED to an outpatient observation unit for a brief stay (<12 hours) prior to discharge home. We corrected their categorization.

eAppendix 7. Defining 7-day Pulmonary Embolism–Related Hospitalization for Those Managed as Outpatients

Our primary safety outcome for those managed as outpatients was 7-day hospitalization for PE-related signs, symptoms, or interventions, defined a priori and used in prior studies in our setting.[13,14] These include complaints of dyspnea, chest pain, syncope or pre-syncope, limb pain or swelling, bleeding or findings of pleural effusion, elevated liver enzyme levels, new anemia or hemorrhage, or new or worsening deep venous thrombosis or PE, or interventions including assistance with medication administration, respiratory support (nonrebreather mask, noninvasive ventilation, intubation or mechanical ventilation), parenteral vasopressor administration, inferior vena cava filter placement or removal, or cardiopulmonary resuscitation. This near-term temporal horizon (7 days) is of concern for treating emergency physicians[15] and encompasses the approximate hospital length of stay for U.S. patients with acute PE.

© 2022 Vinson DR et al. *JAMA Network Open.*

eTable 2. Emergency Department Patients With Acute Pulmonary Embolism Stratified by Direct Discharge Home vs Admission to an Observation Unit or Hospital

Characteristics	Total cohort (N=1039) n (%)	Initial outpatient management		*P* value
		Yes (n=278; 26.7%) n (%)	No (n=761; 73.1%) n (%)	
Age, years, median (IQR)	65 (52-74)	61 (47-70)	67 (56-75)	<.001
Sex				
Female	533 (51.3)	153 (55.0)	380 (49.9)	.15
Male	506 (48.7)	125 (45.0)	381 (50.1)	
Race/ethnicity, self-reported[a]				
African American	150 (14.4)	46 (16.6)	104 (13.7)	.73
Asian	65 (6.3)	16 (5.8)	49 (6.4)	
Hispanic or Latinx	110 (10.6)	30 (10.8)	80 (10.5)	
White	707 (68.1)	185 (66.6)	522 (68.6)	
Other	7 (0.7)	1 (0.4)	6 (0.8)	
Comorbidities				
Chronic lung disease	275 (26.5)	64 (23.0)	211 (27.7)	.13
Cancer (active or history of)	252 (24.3)	56 (20.1)	196 (25.8)	.06
Heart failure (systolic or diastolic)	43 (4.1)	6 (2.2)	37 (4.9)	.05
Arrival by ambulance	188 (18.1)	18 (6.5)	170 (22.3)	<.001
Worst vital signs[b]				
Systolic blood pressure <100 mmHg	162 (15.6)	15 (5.4)	147 (19.3)	<.001
Heart rate ≥110 beats/min	278 (26.8)	28 (10.1)	250 (32.9)	<.001
Respiratory rate ≥30 breaths/min	120 (11.6)	4 (1.4)	116 (15.2)	<.001
Pulse oximetry <90%[c]	140 (13.5)	4 (1.4)	136 (17.9)	<.001
Temperature <36°C	19 (1.8)	2 (0.7)	17 (2.2)	.11
Diagnostic imaging, timing				
Pre-arrival (<12h)	107 (10.3)	57 (20.5)	50 (6.6)	<.001
Emergency department	932 (89.7)	221 (79.5)	711 (93.4)	
Pulmonary Embolism Severity Index Class				
I-II (lower risk)	474 (45.6)	190 (68.4)	284 (37.3)	<.001
III-IV (intermediate risk)	393 (37.8)	74 (26.6)	319 (41.9)	
V (highest risk)	172 (16.6)	14 (5.0)	158 (20.8)	

© 2022 Vinson DR et al. *JAMA Network Open.*

Troponin I concentration[d]				
Normal	630 (60.6)	211 (75.9)	419 (55.1)	<.001
Elevated	263 (25.3)	4 (1.4)	259 (34.0)	
Not measured	146 (14.1)	63 (22.7)	83 (10.9)	

[a] Other race/ethnicity includes Native American and Hawaii and Pacific Islander
[b] Worst in the direction in question measured during the emergency department encounter
[c] With or without oxygen supplementation
[d] We report the highest concentration measured during the emergency department encounter..

© 2022 Vinson DR et al. *JAMA Network Open.*

eTable 3. Characteristics and Course of Emergency Department Patients With Acute Pulmonary Embolism Discharged Directly Home Who Subsequently Experienced 7-Day Hospitalization for Pulmonary Embolism–Related Concerns or 30-Day All-Cause Mortality

Age Sex<br>PESI Score<br>(Class)	Clinical Course
7-day PE-related hospitalization	
27-year-old F<br>PESI: 44 (Class I)	The patient presented to an intervention ED with a chief complaint of tachycardia and mild dysuria for 1 week. Her past medical history was notable for psychosis but without risk factors for venous thromboembolism. Her initial vital signs were normal except for tachycardia, which improved after intravenous fluid treatment, but did not fully resolve. The rest of her physical examination was normal. Urinalysis was negative. CTPA was positive for PE without right ventricular dilatation. She was started on rivaroxaban in the ED and discharged home with a next-day appointment with her primary care physician and a consult to Anticoagulation Management Services. She returned to the ED less than 24 hours later complaining of abdominal pain and chest pain and was admitted to the hospital. She had delusional parasitosis and agitation, requiring intravenous antipsychotics. She remained hemodynamically stable and did not require supplemental oxygen or respiratory support. She was discharged 5 days later to a psychiatric facility and continued rivaroxaban for 6 months without consequence.
53-year-old F<br>PESI: 50<br>(Class I)	The patient presented to a control ED with 1 day of right-sided chest pain that radiated to the right shoulder. She denied dyspnea. History was notable for right knee replacement 10 days prior with twice-daily aspirin for venous thromboembolic prophylaxis. Vital signs were unremarkable, and no leg swelling was noted. CTPA was positive for PE without right ventricular dilatation. Her serum troponin and 12-lead electrocardiogram were negative. Rivaroxaban was prescribed with strict return precautions to the ED. She returned the next day for new-onset dyspnea. Serial vital signs were normal. She felt better and was discharged home with next-day with follow-up with her primary care physician. At that visit she complained of worsening dyspnea, severe pleuritic chest pain, fever, and cough. Her vital signs were normal, except for an elevated temperature. A portable chest radiograph showed a new right pleural effusion and adjacent focal opacities. Her physician consulted an emergency physician, then had the nurse transport the patient by wheelchair on nasal cannula oxygen to the ED on the same campus. A bedside echocardiogram revealed a normal heart and pulmonary findings consistent with pneumonia. She was started on intravenous piperacillin-tazobactam and hospitalized. She improved in house and was discharged on the 3rd hospital day on amoxicillin-clavulanate. She completed 3 months of rivaroxaban without incident.
65-year-old F<br>PESI: 115<br>(Class IV)	The patient presented to an intervention ED with sudden-onset dyspnea on exertion 1 week prior associated with cough. History was notable for metastatic breast cancer, neutropenia from chemotherapy, and prior pleural effusions. Her initial set of vital signs were normal except for mild tachycardia that resolved after intravenous fluid administration. CTPA revealed multiple bilateral PE in the segmental and subsegmental branches, as well as moderate right pleural effusion. There was no right ventricular dilatation. Though a DOAC was offered, she preferred to start enoxaparin in the ED. She was discharged home with enoxaparin and warfarin and returned the next day to medicine clinic for instruction on enoxaparin administration. She also spoke by telephone with Anticoagulation Management Service. She presented to the ED 5 days later for

© 2022 Vinson DR et al. *JAMA Network Open.*

	worsening dyspnea on exertion and several days of fever. Her laboratory results showed therapeutic international normalized ratio as well as influenza B and elevated lactate. She was hospitalized for intravenous cefepime and oseltamivir for severe sepsis. She remained hemodynamically stable throughout her hospital course. She was discharged home on the 4th hospital day on cefpodoxime, doxycycline, and oseltamivir and indefinite warfarin management without incident.
66-year-old M PESI: 76 (Class II)	The patient presented to an intervention ED with 1 week of right-sided pleuritic chest pain and cough. History was notable for hypertension and remote kidney transplant. A D-dimer was found to be elevated and a CTPA was positive for 2 subsegmental, right-sided PEs with a small, right-sided pleural effusion. He was discharged home on rivaroxaban and followed up with Anticoagulation Management Services by telephone the following day. He complained of persistent right-sided chest pain and chills at his primary care physician's office 3 days later. An outpatient chest radiograph showed a new focal consolidation and worsening right-sided pleural effusion. He was sent to the ED, where he was diagnosed with pneumonia and admitted for intravenous antibiotics. During hospitalization, the patient required no supplemental oxygen and was discharged home on the third hospital day on oral antibiotics. He has continued his rivaroxaban indefinitely without sequelae.
30-day all-cause mortality	
98-year-old M PESI: 178 (Class V)	The patient presented to an intervention ED with 2 days of dyspnea and right calf pain. History was notable for hypertension, melanoma, atrial fibrillation, cerebrovascular disease, protein calorie malnutrition and deep vein thrombosis. He was not taking anticoagulation medication at the time due to previous upper gastrointestinal bleeding. He did not want resuscitation. His vital signs were normal, except a chronically low systolic blood pressure. He was cachectic on examination and had no leg swelling or tenderness. D-dimer was found to be elevated and a CTPA revealed 2 subsegmental right-sided PEs. No deep vein thrombosis was identified on compression ultrasonography. In a shared decision-making conversation with the patient and his family, they agreed to restart anticoagulation. Follow-up was arranged with his primary care physician and Anticoagulation Management Services. He was discharged home on enoxaparin and warfarin and spoke with a pharmacist by telephone the next day for anticoagulation management. He presented to the ED 2 weeks later for failure to thrive and was subsequently discharged home with hospice care, where he died 3 days later.
58-year-old M PESI: 98 (Class III)	The patient presented to a control ED with 2 weeks of progressively worsening fever, dyspnea and cough. History was notable for glioblastoma multiforme status post palliative resection and chemotherapy. He was hypoxic in the ED and was placed on supplemental oxygen. A D-dimer returned elevated. CTPA was positive for multiple scattered emboli in both lower lobes and a right lower lobe pulmonary infarct. He declined anticoagulation therapy due to increased bleeding risk and elected palliative measures instead. He was discharged home with hospice care and died 2 days later.

Abbreviations: CTPA, computed tomography pulmonary angiogram; ED, emergency department; PE, pulmonary embolism; PESI, Pulmonary Embolism Severity Index; DOAC, direct oral anticoagulant.

© 2022 Vinson DR et al. *JAMA Network Open.*

eAppendix 8. Thirty-Day All-Cause Mortality of Emergency Department Patients With Acute Pulmonary Embolism

Thirty-day all-cause mortality varied by site of care: it was lower among those discharged home than those hospitalized: 0.7% (n=2) vs 5.7% (n=43) (*P*<.001). We describe the characteristic and course of the 2 outpatients who died within 30 days in **eTable 3** above.

Thirty-day all-cause mortality also varied by risk class. We report mortality stratified by PE Severity Index classification in **eTable 4** below. Thirty-day mortality was similar between ED groups: 4.7% (n=26) in the intervention group and 3.9% (n=19) in the control group. Among patients with complete mortality data, the 30-day mortality was 3.8% and was lower among outpatients than their hospitalized counterparts (0.9% vs 4.8%).

© 2022 Vinson DR et al. *JAMA Network Open.*

eTable 4. Thirty-Day All-Cause Mortality of Emergency Department Patients With Acute Pulmonary Embolism Stratified by the Pulmonary Embolism Severity Index Classification

**Pulmonary Embolism Severity Index Classification**	**Thirty-day all-cause mortality** n (%)
All risk classes (N=1,039)	45 (4.3)
Risk strata	
Classes I-II (lower risk), n=474	2 (0.4)
Classes III-IV (intermediate risk), n=393	16 (4.1)
Class V (highest risk), n=172	27 (15.7)

eAppendix 9. A Large U.S. Study of Claims Data

A large U.S. study of claims data found that outpatient management of insured patients increased from 2011 to 2018 (16% to 23%).[16] This attests to changing practice patterns in some circles, but the results from this study cannot be compared with our own. The initial site-of-care (clinic vs ED) was not reported, and we know that clinic patients with PE are far lower risk than those diagnosed in the ED.[17,18] Also, sicker patients were excluded from their study (e.g., those treated with low molecular weight heparin, like most patients with active cancer, and those with contraindications to anticoagulation). Excluding these higher-risk patients from the study may have inflated the proportion of patients undergoing outpatient management.

© 2022 Vinson DR et al. *JAMA Network Open.*

eReferences

1. Stevens SM, Woller SC, Baumann Kreuziger L, et al. Executive Summary: Antithrombotic Therapy for VTE Disease: Second Update of the CHEST Guideline and Expert Panel Report. *Chest*. 2021;160(6):2247-2259. doi:10.1016/j.chest.2021.07.056
2. Kabrhel C, Rosovsky R, Baugh C, et al. Multicenter Implementation of a Novel Management Protocol Increases the Outpatient Treatment of Pulmonary Embolism and Deep Vein Thrombosis. *Acad Emerg Med*. 2019;26(6):657-669. doi:10.1111/acem.13640
3. Kabrhel C, Rosovsky R, Baugh C, et al. The creation and implementation of an outpatient pulmonary embolism treatment protocol. *Hosp Pract (1995)*. 2017:1-7. doi:10.1080/21548331.2017.1318651
4. Kabrhel C, Vinson DR, Mitchell AM, et al. A clinical decision framework to guide the outpatient treatment of emergency department patients diagnosed with acute pulmonary embolism or deep vein thrombosis: Results from a multidisciplinary consensus panel. *J Am Coll Emerg Physicians Open*. 2021;2(6):e12588. doi:10.1002/emp2.12588
5. Aujesky D, Obrosky DS, Stone RA, et al. Derivation and validation of a prognostic model for pulmonary embolism. *Am J Respir Crit Care Med*. 2005;172(8):1041-6. doi:10.1164/rccm.200506-862OC
6. Aujesky D, Roy PM, Verschuren F, et al. Outpatient versus inpatient treatment for patients with acute pulmonary embolism: an international, open-label, randomised, non-inferiority trial. *Lancet*. 2011;378(9785):41-8. doi:10.1016/S0140-6736(11)60824-6
7. Vinson DR, Ballard DW, Mark DG, et al. Risk stratifying emergency department patients with acute pulmonary embolism: Does the simplified Pulmonary Embolism Severity Index perform as well as the original? *Thromb Res*. 2016;148:1-8. doi:10.1016/j.thromres.2016.09.023
8. Erkens PM, Gandara E, Wells P, et al. Safety of outpatient treatment in acute pulmonary embolism. *J Thromb Haemost*. 2010;8(11):2412-7. doi:10.1111/j.1538-7836.2010.04041.x
9. Zondag W, Mos IC, Creemers-Schild D, et al. Outpatient treatment in patients with acute pulmonary embolism: the Hestia Study. *J Thromb Haemost*. 2011;9(8):1500-7. doi:10.1111/j.1538-7836.2011.04388.x
10. Kearon C, Akl EA, Ornelas J, et al. Antithrombotic Therapy for VTE Disease: CHEST Guideline and Expert Panel Report. *Chest*. 2016;149(2):315-52. doi:10.1016/j.chest.2015.11.026
11. Stevens SM, Woller SC, Kreuziger LB, et al. Antithrombotic Therapy for VTE Disease: Second Update of the CHEST Guideline and Expert Panel Report. *Chest*. 2021;160(6):e545-e608. doi:10.1016/j.chest.2021.07.055
12. Erkens PM, Gandara E, Wells PS, et al. Does the Pulmonary Embolism Severity Index accurately identify low risk patients eligible for outpatient treatment? *Thromb Res*. 2012;129(6):710-4. doi:10.1016/j.thromres.2011.08.025
13. Vinson DR, Ballard DW, Huang J, et al. Outpatient Management of Emergency Department Patients With Acute Pulmonary Embolism: Variation, Patient Characteristics, and Outcomes. *Ann Emerg Med*. 2018;72(1):62-72 e3. doi:10.1016/j.annemergmed.2017.10.022
14. Vinson DR, Mark DG, Chettipally UK, et al. Increasing safe outpatient management of emergency department patients with pulmonary embolism: a controlled pragmatic trial. *Ann Intern Med*. 2018;169(12):855-865. doi:10.7326/M18-1206
15. Kabrhel C, Okechukwu I, Hariharan P, et al. Factors associated with clinical deterioration shortly after PE. Research Support, Non-U.S. Gov't. *Thorax*. 2014;69(9):835-42. doi:10.1136/thoraxjnl-2013-204762
16. Lutsey PL, Walker RF, MacLehose RF, et al. Inpatient Versus Outpatient Acute Venous Thromboembolism Management: Trends and Postacute Healthcare Utilization From 2011 to 2018. *J Am Heart Assoc*. 2021;10(20):e020428. doi:10.1161/jaha.120.020428

© 2022 Vinson DR et al. *JAMA Network Open.*

17. Vinson DR, Hofmann ER, Johnson EJ, et al. Management and Outcomes of Adults Diagnosed with Acute Pulmonary Embolism in Primary Care: Community-Based Retrospective Cohort Study. *J Gen Intern Med*. 2022;doi:10.1007/s11606-021-07289-0
18. Vinson DR, Bath H, Huang J, Reed ME, Mark DG, Network C. Hospitalization Is Less Common in Ambulatory Patients With Acute Pulmonary Embolism Diagnosed Before Emergency Department Referral Than After Arrival. *Acad Emerg Med*. 2020;27(7):588-599. doi:10.1111/acem.14034

© 2022 Vinson DR et al. *JAMA Network Open.*
